# A Novel Redox Method for Rapid Production of Functional Bi-Specific Antibodies For Use in Early Pilot Studies

**DOI:** 10.1371/journal.pone.0022533

**Published:** 2011-07-21

**Authors:** Jennifer Carlring, Evy De Leenheer, Andrew William Heath

**Affiliations:** Academic Unit of Immunology and Infectious Diseases, Department of Infection and Immunity, School of Medicine and Biomedical Sciences, University of Sheffield, Sheffield, United Kingdom; London School of Hygiene and Tropical Medicine, United Kingdom

## Abstract

We demonstrate here a rapid alternative method for the production of functional bi-specific antibodies using the mild reducing agent 2-mercaptoethanesulfonic acid sodium salt (MESNA). Following reduction of a mixture of two monoclonal antibodies with MESNA to break inter heavy chain bonds, this solution is dialysed under oxidising conditions and antibodies are allowed to reform. During this reaction a mixture of antibodies is formed, including parental antibodies and bi-specific antibody. Bi-specific antibodies are purified over two sequential affinity columns. Following purification, bi-specificity of antibodies is determined in enzyme-linked immunosorbent assays and by flow cytometry. Using this redox method we have been successful in producing hybrid and same-species bi-specific antibodies in a time frame of 6–10 working days, making this production method a time saving alternative to the time-consuming traditional heterohybridoma technology for the production of bi-specific antibodies for use in early pilot studies. The use of both rat and mouse IgG antibodies forming a rat/mouse bi-specific antibody as well as producing a pure mouse bi-specific antibody and a pure rat bi-specific antibody demonstrates the flexibility of this production method.

## Introduction

Köhler and Milstein [1] pioneered hybridoma technology and thereby opened the possibility to manufacture pure monoclonal antibody (mAb) in large amounts. MAbs are not generally efficient on their own as immunotherapeutic agents and have therefore been attached or conjugated to more potent agents including toxins, radionucleotides and cytotoxic drugs. Whereas mAbs are specific for one epitope, bi-specific antibodies (bsAb) are able to recognize two epitopes on the same or a distinct antigen simultaneously. Although bsAbs have attracted attention as candidates for cancer therapy [2], they have encountered obstacles including improper heterodimer formation and low yields [3], [4]. Traditionally, bsAbs have been produced using hybrid hybridoma technology [5], which relies on time-consuming tissue culture methodology. Additionally, co-expression of two immunoglobulin G (IgG) molecules in a hybrid hybridoma can produce up to 10 different heavy and light-chain pairings leading to a low yield of the required bi-specific antibody [6]. Finally, separation of bsAb from other immunoglobulins in supernatant can be very difficult, particularly when the two component mAbs are from the same species and subclass. Another approach taken in order to produce bsAbs involves chemical conjugation of two antibodies or two antibody fragments [7]. Problems using this method include the inactivation, unfolding or aggregation of the bsAb due to the chemical conditions used during the production. A more recent approach taken by several researchers involves the use of molecular means to produce a range of bsAb including: single chain variable fragment (scFv) fusions or diabodies, scFv Fc fusions and single variable domain IgGs as well as dual-variable domain IgG [8]–[11].

**Figure 1:**
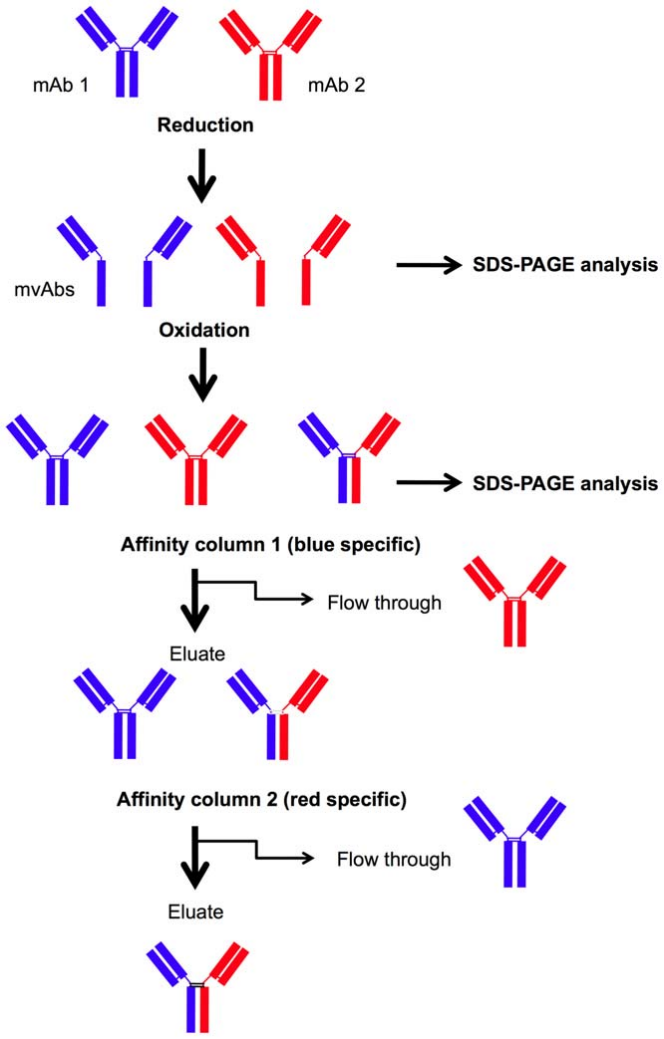
Redox method overview. MAb 1 and mAb 2 are mixed and reduced using MESNA, resulting in mvAb fragments being formed as visualized by SDS-PAGE. When dialysed into oxidising conditions mAbs are reformed resulting in a mixture of parental and bi-specific antibodies. In order to get a pure bsAb solution, antibodies are purified over two sequential affinity columns, each one specific for the one mAb only. After affinity column one the eluate containing mAb 1 and bsAb is purified on column 2. The resulting eluate contains pure bsAb that can be used for further applications.

We have developed a chemical reduction-oxidation (redox) method for the production of purified bsAbs in a fraction of the time taken by the traditional hybrid hybridoma technology by using the mild reducing agent 2-mercaptoethanesulfonic acid sodium salt (MESNA) followed by dialysis under oxidising conditions in order to allow antibodies to reform. During this reaction a mixture of antibodies is formed, including parental antibodies and bi-specific antibody. Bi-specific antibodies are highly purified over two sequential affinity columns. We show here the production of several different bsAbs that have been purified to homogeneity using affinity columns. A simplified schematic overview of this novel redox method can be seen in Figure 1. To demonstrate that it is possible to make bsAbs using mAbs from different species we have made rat/mouse hybrid bsAbs and purified these, first over an anti-rat IgG and secondly an anti-mouse IgG affinity column. In addition to this we demonstrate that it is possible to make bsAbs using two different antibodies from the same species and subclass. In order to purify these bsAbs the parental antibodies were conjugated to biotin or dinitrophenol prior to reduction using MESNA. Purification of bsAbs was carried out by sequential purification on anti-biotin and anti-DNP affinity columns. All bsAbs produced have the ability to simultaneously bind two antigens and show functionality *in vitro* as demonstrated by enzyme linked immunosorbent assay (ELISA) and by flow cytometry.

## Materials and Methods

### Monoclonal antibodies

Antibodies used were rat anti-β-galactosidase mAb (clone GL117, IgG2a) [12], rat anti-mouse CD40 mAbs (clone 1C10, IgG2a and clone 10C8, IgG1) [12], mouse anti-human CD40 mAb (clone G28/5, IgG2a) [13] and mouse A20 IgG mAb (IgG2a) [14]. A20 IgG is a tumor idiotype and is expressed on a BALB/c B cell lymphoma originally derived from a spontaneous reticulum cell neoplasm. The specificity of the A20 mAb is unknown.

### Redox methodology

100 mM 2-mercaptoethanesulfonic acid sodium salt (MESNA; Sigma-Aldrich, Gillingham, UK) was used to reduce mAb to monovalent antibody (mvAb) by adding an equal volume of two times concentrated MESNA solution in dH_2_O [15] to a mixture of 1 mg mAb 1 and 1 mg of mAb 2 in PBS and incubated at 37°C for 25 minutes. Thereafter mvAbs were exposed to oxidising conditions by dialysis against three buffer exchanges of phosphate buffered saline (PBS), pH 7.4 over 24 h at 4°C. Bi-specific antibody was purified from parental antibody over sequential affinity columns to assure only pure bsAb was assayed. The eluted fraction from column one was dialyzed against PBS and subsequently purified over column two with the eluted fraction from this column containing bsAb. BsAb concentration was determined using a micro BCA protein assay kit (Thermo Scientific, Rockford, USA).

### Hybrid bsAb production and purification

For production of hybrid bsAbs 1 mg of GL117 mAb and 1 mg of A20 mAb or 1 mg of 1C10 mAb and 1 mg of A20 mAb were reduced with MESNA as described above. Bi-specific antibody was purified from parental antibody over affinity column one (GE Healthcare, St Giles, UK), coupled with affinipure goat anti-rat IgG (minimum cross reaction with mouse, human, bovine, horse and rabbit serum proteins; Jackson ImmunoResearch Europe Ltd, Newmarket, UK). Antibody mixture was applied manually ‘drop to drop’ using a syringe at a flow rate of 1 ml/min. Bound antibody was eluted using 0.1 M Glycine-HCl, pH 2.7 and the pH of the eluate was neutralized by the addition of a 1/10 dilution 1.0 M Tris-HCl, pH 9.0. The eluted fraction from the anti-rat column was dialyzed against three changes of PBS and subsequently purified over an affinipure goat anti-mouse IgG (minimum cross reaction with rat, human, bovine, horse and rabbit serum proteins; Jackson ImmunoResearch), as described for the anti-rat column with the eluted fraction from this column containing bsAb.

### Screening ELISA 1 – recognition of hybrid bsAb by anti-rat and anti-mouse heavy chain antibodies

Ten µg/ml goat anti-rat IgG (AbD SeroTec, Kidlington, UK) in PBS was adsorbed to 96-well ELISA plates for 16 h at 4°C. In all ELISA assays described the incubation steps were at room temperature for 1 hour and following each incubation step plates were washed with PBS +0.05% Tween 20, and plates blocked with 5% (v/v) bovine serum albumin (BSA; Sigma Aldrich) unless specified otherwise. Samples were added at a two-fold dilution from the neat sample and further two fold serial dilutions of bsAb and parental mAbs in PBS were added to the plates. Plates were incubated with peroxidase conjugated polyclonal anti-mouse Ig (multiple adsorbed; BD Biosciences, Oxford, UK). Substrate (o-phenylenediamine dihydrochloride; Sigma Aldrich) was added and following colour development absorbance read at 450 nm using an ELISA plate reader (EL_X_808, Bio-Tek Instruments Inc., Potton, UK).

### Screening ELISA 2 - Binding of hybrid bsAb to β-galactosidase and simultaneous recognition by anti-mouse IgG heavy chain

Recombinant purified β-gal protein (Alpha Laboratories, Eastleigh, UK) at a concentration of 5 µg/ml in PBS was adsorbed to 96-well ELISA plates for 16 h at 4°C. The assay was finished as described for assay 1.

### Screening ELISA 3 – Binding of hybrid bsAb mouse CD40 and simultaneous recognition by anti-mouse IgG heavy chain

Ten µg/ml anti-human IgG (Fc specific; Sigma Aldrich) in PBS was adsorbed to 96-well ELISA plates for 16 h at 4°C. Recombinant mouse CD40/HuFc chimera (R&D Systems Europe Ltd., Abingdon, UK) was added at 0.1 µg/ml in PBS for 1 h at room temperature. The assay was finished as described for assay 1.

### Determination of functionality of hybrid bsAbs using flow cytometry

CD40L cells are stably transfected murine L929 fibroblast cells that express murine CD40 on their cell surface [16]. These cells were used to ensure rat anti-mouse CD40 hybridised with mouse IgG was able to bind to native CD40 *in vitro.* Cells were prepared for cell surface flow cytometric analysis and incubated with 1–5 µg hybrid bsAb and relevant controls for 30 min on ice in PBS +0.1% (v/v) BSA. Cells were washed twice by centrifugation at 400 x *g* for 5 min. To detect staining, CD40L cells were incubated with a secondary anti-mouse IgG-FITC mAb (BD Biosciences) for 30 min on ice in PBS +0.1% (v/v) BSA. Following two centrifugation steps in PBS +0.1% (v/v) BSA cells were analysed on a FACSCalibur (BD Biosciences) or an LSRII (BD Biosciences). TO-PRO®-3-iodide (Invitrogen Ltd, Paisley, UK) was added immediately prior to analysis at a final concentration of 0.3 µM to allow dead cell exclusion. Data analysis was carried out using FlowJo™ software (Tree Star Inc., Ashland, USA).

### Production of same species bsAbs

In order to be able to purify bi-specific antibodies made from the same species and antibody subclass, parent antibodies were pre-labeled with biotin or DNP prior to redox exposure.

### Antibody biotinylation

Antibody was buffer exchanged into 0.1 M NaHCO_3_ using dialysis prior to biotinylation. (+)-Biotin-N-hydroxysuccinimide ester (Sigma Aldrich) was dissolved in DMSO (Sigma Aldrich) to give 10 mg/ml and 80 µg of biotin was added per mg of antibody and incubated for 4 hours at room temperature in the dark during continuous rotation on a MACSMix (Miltenyi Biotech Ltd., Bisley, UK). Biotinylated antibody was buffer exchanged into PBS pH 7.4, using dialysis over 24 h at 4°C and thereafter used in further applications.

### Haptenation of antibodies using DNFB

Dinitrophenol was conjugated to mouse A20 IgG mAb as previously described [17]. Briefly, 1-fluoro-2,4-dinitrobenzene (DNFB; Sigma Aldrich) was diluted in chloroform to 50 mg/ml. Mouse or rat mAb at 10 mg/ml in 0.1 M NaHCO_3_ was combined with DNFB at a working concentration of 0.5 mg/ml and incubated at 37°C for 45–60 minutes in the dark during continuous rotation on a MACSMix. The haptenised antibody was subsequently dialysed into PBS over 24 h at 4°C and used in further applications.

### Production and purification of same species bsAb

For production of a mouse bsAb 1 mg of DNP labeled G28/5 mAb and 1 mg of biotinylated A20 mAb (both mouse IgG2a) were mixed. Additionally, for the production of a rat bsAb 1 mg DNP labeled 1C10 mAb and 1 mg biotinylated GL117 mAb (both rat IgG2a) were mixed and incubated with MESNA as described above. Following dialysis into PBS bivalent antibody was purified from parental antibody over Amino-link plus affinity columns (Thermo Scientific). Column one was coupled with anti-biotin antibody (Thermo Scientific) following the manufacturers'instructions. The eluted fraction from the anti-biotin column was dialysed against PBS and subsequently purified over a column coupled with anti-DNP antibody (Bethyl Laboratories, Universal Biologicals Ltd, Cambridge, UK), with the eluted fraction from this column containing bsAb.

### Screening of same species bsAb using ELISA

To determine that antibody labeling had been successful and that same species bsAb had been generated two ELISA assays were designed.

### Determination of DNP and biotin presence on bsAb

Goat anti-DNP antibody was adsorbed to 96-well ELISA plates at 2 µg/ml in PBS for 16 h at 4°C. The ELISA was performed as described for assay 1 using streptavidin horseradish peroxidase (Vector Laboratories Inc., Peterborough, UK) at 0.5 µg/ml for detection.

### Binding to human CD40 and detection by SA-HRP simultaneously

Recombinant human CD40/HuFc was directly adsorbed to 96-well ELISA plates at 5 µg/ml in 0.1 M sodium carbonate buffer for 16 h at RT. The ELISA was performed as described for assay 1 using streptavidin horseradish peroxidase (Vector Laboratories) at 0.5 µg/ml as the detection antibody.

## Results and Discussion

We have successfully produced several bsAbs using this redox technology, including hybrid rat/mouse and same species bsAbs. Hybrid rat/mouse bsAbs include GL117-A20 bsAb and 1C10-A20 bsAb and same species bsAbs include mouse G28/5-A20 bsAb and rat 1C10-GL117 bsAb. For simplicity most of the optimisation results shown are for the GL117-A20 bsAb, however the redox production method was identical for all bsAbs, with the exception of the use of different affinity columns for purification purposes of hybrid and same species bsAbs.

**Figure 2:**
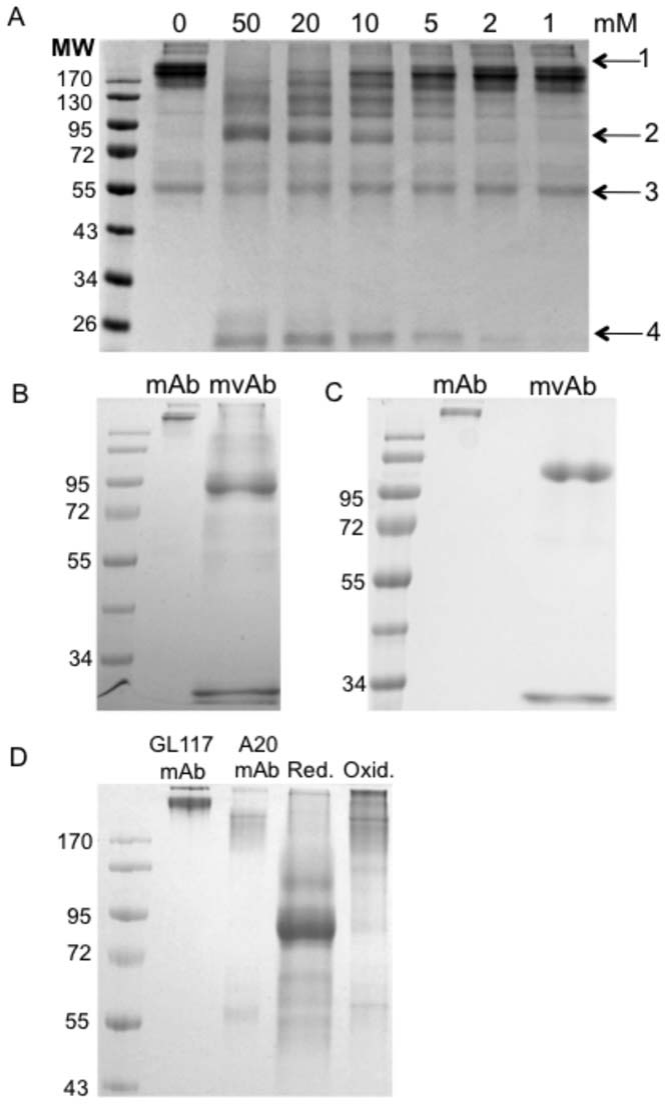
Redox method optimisation. Non-reducing SDS-PAGE showing the range of MESNA concentrations used in optimisation studies for GL117 mAb (a). Arrows indicate presence of whole mAb (1), mvAb (2), heavy chain (3) and light chain (4). 50 mM MESNA efficiently cleaves inter heavy chains in GL117 mAb resulting in a band at around 90 kDa (b) and in A20 mAb at around 95 kDa (c). Dialysis into PBS shows successful reformation of mvAb into whole mAb (d). Image shows in lane order; molecular weight standard, parental GL117 mAb and parental A20 mAb (both >170 kDa), MESNA-reduced GL117 and A20 mvAb mixture (90–110 kDa) and reformed whole antibody, including bi-specific GL117-A20 bsAb. Molecular weight (MW) marker PageRuler pre-stained protein ladder 10–170 kDa (Fermentas GmbH, St. Leon-Rot, Germany). Red.  =  Reducing conditions, Oxid.  =  Oxidising conditions.

To optimise reduction conditions an initial time course experiment was carried out. Following analysis of samples reduced by MESNA by sodium dodecyl sulfate polyacrylamide gel electrophoresis (SDS-PAGE) under non-reducing conditions it was determined that incubation at 37°C for 25 minutes was optimal (data not shown). To further optimise reduction conditions for each antibody MESNA was added to a final concentration of 50, 20, 10, 5, 2, and 1 mM to the antibody of choice. Figure 2a shows the range of MESNA concentrations used for the reduction of A20 mAb to mvAb. The optimum MESNA concentration for use was determined to be 50 mM for A20 mAb and GL117 mAb and 20 mM for 1C10 mAb and G28/5 mAb, as at these concentrations very small amounts of whole antibody is still present. All antibodies used in this study were effectively reduced to mvAb at their optimum MESNA concentration. As can be seen in Figure 2a, reduction with MESNA results in the release of some free heavy and light chain. Efficient mvAb formation yielded a band at a molecular weight ranging from 72 to 110 kDa, depending on antibody used. Figures 2b and 2c further illustrate the efficient cleavage of the inter-heavy chain disulfide bonds in GL117 and A20 antibodies after reduction with 50 mM MESNA resulting in mvAbs of approximately 90 kDa and 110 kDa, respectively.

The successful reformation of the inter-heavy chain disulfide bonds of A20 mvAb and GL117 mvAb following dialysis in PBS was visualized using non-reducing SDS-PAGE and is shown in Figure 2d. The loss of the band at 90–110 kDa verifies reformation of whole antibody molecules. After oxidation, the free light chain is no longer present indicating that this is reformed with the heavy chain to produce whole antibody molecules.

**Figure 3:**
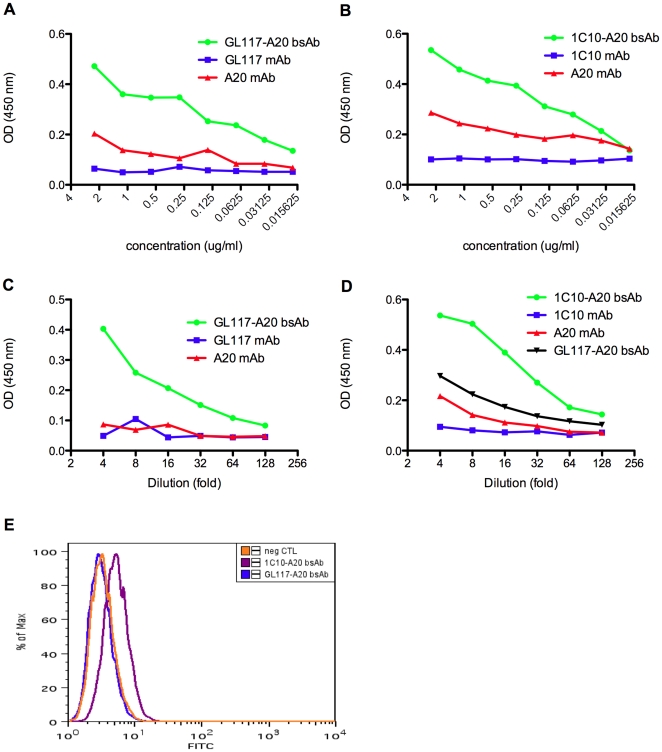
Verification of antigen recognition by hybrid bsAbs. The presence of GL117-A20 bsAb (a) and 1C10-A20 bsAb (b) was verified by simultaneous recognition of anti-rat IgG and anti-mouse IgG in ELISA assay 1 as visualized by an increase in the OD reading at 450 nm compared to parental mAbs. Antigen recognition of β-gal and recognition by an anti-mouse IgG antibody was determined in ELISA assay 2 showing the presence of hybrid GL117-A20 bsAb after binding of the GL117 binding arm to β-gal and the A20 binding arm to anti-mouse IgG (c). CD40 specificity and subsequent recognition by an anti-mouse IgG antibody was determined in ELISA assay 3 and this showed the presence of 1C10-A20 bsAb by the binding of the 1C10-binding arm to CD40 and the A20 binding arm to anti-mouse IgG (d). Flow cytometric analysis on viable CD40L cells showed an increase in median fluorescence after incubation with 1C10-A20 bsAb but not GL117-A20 bsAb (e). Neg CTL  =  negative control.

Screening of hybrid bsAb was by ELISA and flow cytometry as described in the methods section. Screening of the hybrid rat/mouse bi-specific antibody was initially based on the recognition of mouse and rat antibodies simultaneously using ELISA assay 1 and this is shown in Figure 3. As only bsAb, and not the parental antibodies, is recognized by anti-mouse capture and anti-rat detection antibodies simultaneously, this assay verifies the presence of bsAb. The superior binding ability of both GL117-A20 bsAb and 1C10-A20 bsAb compared to their parental counterparts is shown in Figure 3a and 3b respectively.

The *in vitro* functionality (antigen binding) of GL117-A20 bsAb was verified in ELISA assay 2 in which recombinant β-gal was bound to the plate. Figure 3c shows binding of the bsAb only, and not of parental antibodies, to β-gal and recognition by anti-mouse antibody simultaneously, providing further evidence that functional bsAb was produced.

To check functionality of the second hybrid rat/mouse bsAb 1C10-A20 its binding to mouse CD40 via the rat IgG component was verified by ELISA using assay 3 and by flow cytometry on CD40L cells. Figure 3d shows binding of 1C10-A20 bsAb to mouse CD40 and the simultaneous detection by an anti-mouse IgG antibody in the ELISA. Despite some non-specific background binding in this assay, we showed that the recognition by 1C10-A20 bsAb of mouse CD40 was specific as determined by flow cytometry using CD40 expressing L929 fibroblast cells. The addition of TO-PRO®-3-iodide prior to analysis allowed the exclusion of dead cells and the subsequent gating on live cells only. The flow cytometry overlay plot in Figure 3e shows that GL117-A20 bsAb, which is specific for β-gal, does not bind to live CD40L cells; however, the 1C10-A20 bsAb including the anti-CD40 binding arm is able to bind to these cells.

**Figure 4:**
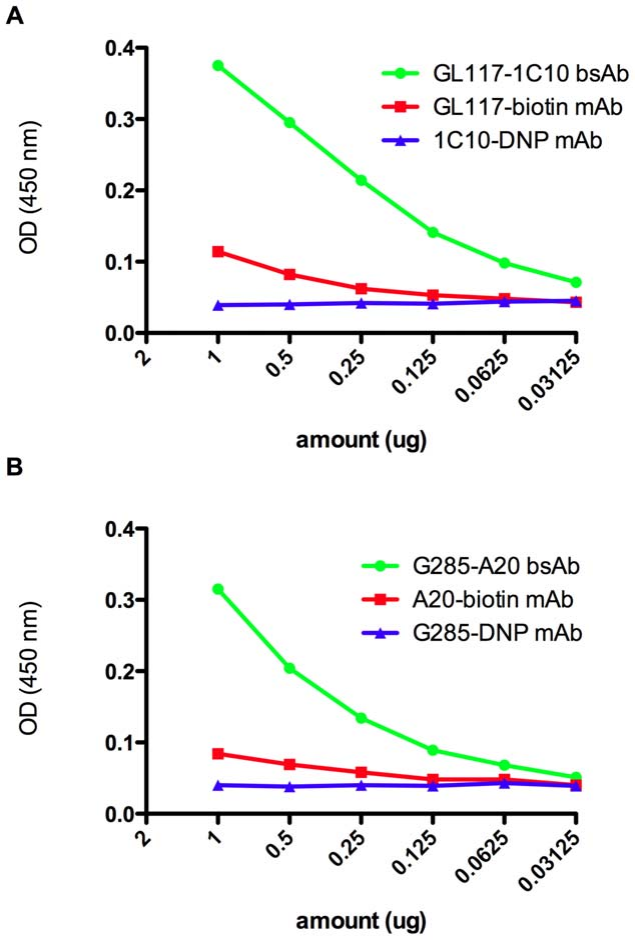
Verification of antigen recognition by same species bsAbs. Same species bsAb was detected using a sandwich ELISA specific for biotin or DNP. Pre-labeling of G28/5 and 1C10 with DNP and A20 and GL117 with biotin allowed binding of 1C10-GL117 bsAb and G28/5-A20 bsAb and not parental antibodies in this assay (a and b respectively).

In addition to the production of hybrid rat/mouse bsAbs, same species mouse and rat bsAbs were made. In order to be able to purify bi-specific antibodies made from the same species, the component antibodies were pre-labeled with biotin or DNP. Following redox the labeled bsAbs were purified over an anti-biotin column followed by an anti-DNP column. To ensure bsAbs were produced these were screened using an anti-biotin and anti-DNP ELISA. Only bsAb carrying the dual labels will be positive in this assay. Figure 4a shows the successful generation of the rat 1C10-GL117 bsAb and Figure 4b a G28/5-A20 mouse bsAb. This data illustrates that it is possible to make bsAb after pre-conjugation to biotin and DNP.

We have shown here that this redox method can be used to produce antigen binding bi-specific antibodies both from different species and most importantly using mAbs of the same subclass and same species. In order to purify these same species bsAbs we labeled the starting material with biotin or DNP. Biotin is one of the eight essential vitamins that comprise the B complex; it is non-toxic and is not known to have any side effects in humans [18]. DNP, being a phenolic compound with potential carcinogenicity would not be ideal for clinical use, but there is a vast array of potential safe haptens available for this purpose. We have simply used DNP as a label in this study to prove in principle that this method works. Production and purification of bsAbs from same species and same subclass starting material using the redox method has in this study been achieved within 3–4 days, Determination of the bsAb specificity and functionality by ELISA and flow cytometry was carried out over the subsequent 3–6 days making the total time frame of production 6–9 days. To produce a bsAb using conventional heterohybridoma methods within the same time frame could be extremely difficult and therefore the use of the redox method will be useful in early pilot studies.

Nineteen percent of the antibody recovered following purification on affinity columns (eluate and flow through) bound to both the columns and was therefore bi-specific. Parental mAb that does not bind to the affinity column can be re-used and exposed to the redox method again potentially increasing the bsAb yield. Although the yield from the redox method is not higher than from the use of traditional methods, it may be sufficient to provide enough material for single patient use. Bargou *et al.* have demonstrated that low amounts of bsAb may be sufficient for successful cancer therapy. The bi-specific T cell engager (BiTE), blinatumomab, which has dual specificity for CD19 and CD3, was used to treat non-Hodgkin' B cell lymphoma. Results from this clinical trial showed tumor reduction after four weeks of daily bsAb immunisations of doses from 0.0005 to 0.6 mg/m^2^. The most effective dose was found to be 0.015 mg/m^2^, indicating that for a potent antibody very small amounts of bsAb may be effective for cancer immunotherapy [19].

In conclusion, we have produced several different bsAb molecules of different specificities using the redox technology. This methodology is widely applicable as it can be used for any two mAbs potentially producing any bsAb of choice and has proved for us to be a successful rapid alternative to the traditional heterohybridoma technology for the production of bi-specific antibodies thereby providing a timesaving solution for early pilot studies.
